# Improving the Anti-Toxin Abilities of the CMG2-Fc Fusion Protein with the Aid of Computational Design

**DOI:** 10.1371/journal.pone.0104674

**Published:** 2014-08-07

**Authors:** Yongyi Xi, Xiaojie Wu, Lihua Gao, Yong Shao, Hui Peng, Hongxing Chen, Huipeng Chen, Xianwen Hu, Junjie Yue

**Affiliations:** Beijing Institute of Biotechnology, Beijing, China; Cincinnati Childrens Hospital Medical Center, United States of America

## Abstract

CMG2-Fc is a fusion protein composed of the extracellular domain of capillary morphogenesis protein 2 (CMG2) and the Fc region of human immunoglobulin G; CMG2-Fc neutralizes anthrax toxin and offers protection against *Bacillus anthracis* challenge. To enhance the efficacy of CMG2-Fc against anthrax toxin, we attempted to engineer a CMG2-Fc with an improved affinity for PA. Using the automatic design algorithm FoldX and visual inspection, we devised two CMG2-Fc variants that introduce mutations in the CMG2 binding interface and improve the computationally assessed binding affinity for PA. An experimental affinity assay revealed that the two variants showed increased binding affinity, and *in vitro* and *in vivo* toxin neutralization testing indicated that one of these mutants (CMG2-Fc(E117Q)) has superior activity against anthrax toxin and was suitable for further development as a therapeutic agent for anthrax infections. This study shows that the computational design of the PA binding interface of CMG2 to obtain CMG2-Fc variants with improving anti-toxin abilities is viable. Our results demonstrate that computational design can be further applied to generate other CMG2-Fc mutants with greatly improved therapeutic efficacy.

## Introduction

Anthrax toxin, which is secreted by *Bacillus anthracis*, is the major virulence factor associated with anthrax infection. Anthrax toxin comprises three proteins: protective antigen (PA), lethal factor (LF), and edema factor (EF) [1], [2]. PA and LF comprise the lethal toxin (LeTx), and PA and EF comprise the edema toxin. PA is critical for the entry of anthrax toxin into cells, where it binds to cellular receptors and transports LF/EF into the cytosol.

Two anthrax toxin receptors have been identified: tumor endothelial marker 8/anthrax toxin receptor 1 (TEM8/ANTXR1) [3], [4] and capillary morphogenesis protein 2/anthrax toxin receptor 2 (CMG2/ANTXR2) [5], [6]. TEM8 is primarily expressed in cancer and embryonic tissues, suggesting that it is related to tumor and embryonic development [7]. CMG2 is widely expressed in human tissues [6], including the heart, lungs, liver, skeletal muscle, small intestine, kidneys, colon, and spleen. The two anthrax receptors are type I transmembrane proteins with three domains: an N-terminal, extracellular von Willebrand factor type A domain (vWA domain), which binds to PA; a single transmembrane spanning domain; and a C-terminal cytosolic domain [8]. Although the vWA domains of the anthrax toxin receptors share a sequence identity of approximately 55%, their affinities for PA binding are remarkably different; CMG2 binds to PA approximately 1,000-fold more potently than TEM8 does (in 1 mM Ca^2+^, the K_D_s are 0.78 nM and 130 nM, respectively) [9]. The widespread expression of CMG2 and its high affinity toward PA imply that CMG2 is the major receptor mediating lethality in the pathogenesis of anthrax; this was also supported by the lethality observed in CMG2-null mice challenged with *B. anthracis* spore infection [10].

Inhibiting the binding of PA to the CMG2 receptor has been a major focal point in developing an effective treatment for anthrax [9]. Many molecules targeting CMG2 have been designed to protect against *B. anthracis* challenge, including antibodies [11], soluble CMG2 [9], [12], [13], and a CMG2-Fc fusion protein [13]–[15]. Soluble CMG2 is the extracellular domain of CMG2 and can effectively neutralize mutant forms of PA that were not neutralized by anti-PA monoclonal antibodies [12]. CMG2-Fc is a fusion protein comprised of soluble CMG2 fused to the human immunoglobulin Fc fragment; this fusion retains the ability to bind to the PA ligand but has a longer circulating half-life and enhances therapeutic effects [13], [14].

A high target affinity is a critical issue for the therapeutic capacity of an inhibitor [16]. Structure-based modeling is one of the common approaches for achieving the goal of improving protein-protein binding affinity [17]. However, it is extremely complicated to select candidate positions for mutation. In the ideal case where protein-protein complex structures are available, the determination of contact residues is straightforward, and this information can be applied to guide the mutation process [18], [19].

In recent decades, a striking series of advances in our knowledge of the three-dimensional structures of anthrax toxins and their complexes with receptors have enabled a greater understanding of the structure-activity relationship of PA [20]–[23]. These studies have provided the opportunity to find molecules with improved abilities to neutralize LeTx. To improve the ability of CMG2-Fc to neutralize anthrax toxin, we focused on elucidating the structure-function relationship of the CMG2-PA complex and identifying the key residues within CMG2 that are responsible for binding PA by investigating the three-dimensional model of the PA-CMG2 complex. We found that the mutation of CMG2 at Glu117 or Tyr158 may decrease the repulsive force between CMG2 and PA, and this reduction should facilitate the interaction between these factors. Based on the above results, we designed two mutants, CMG2 (E117Q)-Fc and CMG2 (Y158Q)-Fc.

A promising mutant, CMG2 (E117Q)-Fc, was identified. The results in this paper show that the protective potency of this mutant in a rat model of anthrax toxin is superior to non-mutant CMG2-Fc. The use of CMG2-Fc (E117Q) for neutralizing anthrax toxin may be a prospective means to treat anthrax in the future. Our results demonstrate the capability for improving protein-binding affinity using computational design. This work will lead to the generation of other optimized CMG2-Fc fusion proteins in future research.

## Materials and Methods

### Ethics statement

Animal experiments were conducted in accordance with the recommendations of the Guide for the Care and Use of Laboratory Animals of the National Institutes of Health and approved by the Animal Ethics Committee of the Academy of Military Medical Sciences (AMMS).

### Computational design of variants using FoldX

To investigate the relationship between the affinity of CMG2-Fc for PA and its ability to neutralize anthrax toxin, a model of CMG2-PA complex was required. The X-ray crystal structures of CMG2 in complex with PA have been solved at a resolution of 2.5 Å [22] and 4.3 Å [23], respectively. These structural models provide valuable information about the interaction between CMG2 and PA.

Because it is not easy to decide which mutations will improve the affinity between subunits in a complex, one possible approach is to ask the FoldX protein design algorithm [24]–[26] to scan all the positions along the interface between the two molecules. In this study, the computational design of high-affinity CMG2 mutants was performed using FoldX. First, the residues comprising the PA-binding interface of CMG2 were identified by using the AnalyseComplex option of FoldX. Next, the residues in the PA-binding interface of CMG2 were scanned by PositionScan to all other 19 naturally occurring amino acids. The effect on the binding energy between CMG2 and PA was calculated as the difference between the binding energy of the mutant and the wild-type amino acid (ΔΔG; in kcal/mol). The amino acid substitutions that caused a decrease in the binding energy toward the PA molecule were selected. PositionScan mutates one amino acid to the other ones and repairs the neighboring residues. This command first mutates the selected position to Ala and annotates all the neighboring residues, then restores the WT residue, and then restores the neighbors to the original amino acids [24]–[26]. A detailed description of the protein design algorithm FoldX (version 3.0) is available elsewhere at http://foldx.crg.es.

Protein structure analysis was performed in Discovery Studio 2.5 (Accelrys, USA). Molecular structure illustrations were generated with PyMOL Molecular Graphics Software (www.pymol.org). The coordinates (PDB ID: 1t6b ) for the CMG2-PA complex structure were taken from the protein data bank.

### Genetic engineering of recombinant CMG2-Fc mutants

According to the preserved DNA sequence of CMG2-Fc (amino acids 1–225 of CMG2 fused to the Fc fragment of human IgG1), we designed two pairs of primers for the introduction of site-specific mutants and one pair of primers for amplification of the full-length CMG2 gene (Table 1). The PCR products were gel extracted and then sub-cloned into the preserved pIRES2-EGFP-Fc vector using NheI and BamHI restriction enzymes. The sequences of the CMG2-Fc mutants were verified prior to plasmid transfection.

**Table 1:** The primers for engineering the mutated constructs.

Name	Sequences(5′–3′)
CMG2-F	CTAGCTAGCCCACCATGGTGGCG
CMG2-R	CGCGGATCCACTTACCTGTCTGCA
117mut-U	CAGTCCCTCGTGAATGTAGGTCTGGCCCACTGGAGACACTCGTTT
117mut-D	CAGACCTACATTCACGAGGGACTG
158mut-U	GGAAATTTTTGCCTCCTTTTCAGCCTGGGAGGGCACCAGTCCATCCA
158mut-D	CAGGCTGAAAAGGAGGCAAAAATTTCC

### CMG2-Fc mutant transfection of Chinese hamster ovary S (CHO-S) cells

The CHO-S cell line for the expression of the CMG2-Fc mutant protein was cultured in a 24-well plate using Dulbecco’s modified Eagle’s medium/F12 (DMEM/F12, Hyclone, USA) containing 10% fetal calf serum (FCS, Hyclone). The CHO-S cells were transfected with the pIRES2-EGFP-Fc vector containing the CMG2 mutant gene using Lipofectamine 2000 (Invitrogen, Carlsbad, CA, USA) according to the manufacturer’s recommended protocols. G418 (0.75 mg/mL) was added to the culture medium for the selection of positive cell colonies (those expressing the CMG2-Fc mutant).

### Purification and isolation of recombinant protein

The CMG2-Fc mutant protein was purified using rProtein A affinity chromatography (GE, Piscataway, NJ, USA) according to the manufacturer’s recommended protocol and eluted with 100 mM Gly-HCl buffer (pH 3.0). The collected fractions were quantified using a BCA Protein Assay (Merck, Darmstadt, Germany), identified by SDS-polyacrylamide gel electrophoresis (under reducing and non-reducing conditions), and visualized by Coomassie G250 staining and Western blot analysis. The target protein was further analyzed by isoelectric focusing electrophoresis and mass spectrometry.

### Binding kinetics of the CMG2-Fc mutants and PA

The affinity of the CMG2-Fc mutant for PA was measured using an Octet QK^e^ system (ForteBio, Menlo Park, CA, USA), a bio-layer interferometry-based instrument that monitors label-free bio-molecular interactions in real time [27]. In brief, the anti-human IgG Fc sensor capturing CMG2-Fc (25 µg/mL) was soaked in PA solutions of various concentrations (in TBS buffer with 2 mmol/L MgCl_2_) to associate with the PA molecule and then soaked in TBS buffer with 2 mmol/L MgCl_2_ to dissociate the PA molecule. The whole course of bio-layer interferometry was monitored. The data were analyzed with ForteBio data analysis software. The experiment parameters were as follows: activation for 30 s, loading for 300 s, washing for 30 s, baseline measurement for 120 s, association for 400 s, and dissociation for 800 s.

### *In vitro* toxin neutralization assay (TNA)

The neutralization of the anthrax toxin was verified using an *in vitro* J774A.1 mouse macrophage cell lysis inhibition assay as described previously [9]. Briefly, the cells were grown in 96-well tissue culture plates (Greiner, Bahlingen, Germany) to approximately 80% confluence. Gradient dilutions of CMG2-Fc (mutant and non-mutant) proteins were added to the cells, followed by a constant concentration of anthrax toxin (200 ng/mL [2.4 nM] PA +200 ng/mL [2.2 nM] LF; Merck). The culture plates were incubated for 2 h at 37°C, 10 µL of CCK8 (Dojindo, Tokyo, Japan) solution was added to each well, and the plates were incubated for a further 2 hours. The absorbance at 450 nm was measured using a microplate reader (Bio-Rad, Hercules, CA, USA).

### Rat LeTx challenge

Rat LeTx challenge experiments were performed in accordance with the guidelines for the treatment of experimental animals in China. Forty male Fisher 344 rats (160–220g) were randomized into eight groups (five rats in every group). Male Fisher 344 rats were inoculated with the LeTx mixture (100 µg PA and 100 µg LF per rat) via the tail vein. For the rats that received the CMG2-Fc mutant and LeTx, a varying amount of mutant was added to the LeTx mixture in a total volume of 400 µL prior to injection. All of the rats were then observed for 24 hours.

### Statistical analysis

We used ForteBio data analysis v6.3 software to analyze the kinetics assays. All the results are reported as the average of three independent trials, and the standard deviation (SD) was calculated in Microsoft Excel 2010. The statistical analysis of all parameters was conducted using Student’s unpaired *t*-tests (SPSS version 11.5.0). For the TNA assay, the effective inhibitory concentrations (EC50s; the concentrations of antitoxin at which 50% of toxin activity is neutralized) were calculated using nonlinear regression analysis.

## Results

### Computational Design of Variants

The X-ray crystal structure of CMG2 in complex with PA was used as a template to design variants of CMG2-Fc that bind to PA with a high affinity. The residues that comprise the CMG2 interface were considered for computational screening. Every selected amino acid position was mutated to all other residues using the PositionScan command of FoldX software. A set of predicted energetic values for CMG2-PA complex formation was obtained and compared with the wild-type CMG2 values. The results of PositionScan showed that amino acid substitutions at CMG2 positions 117 and 158 would cause a decrease in the binding energy with PA (Table 2). Mutations at these positions might favor CMG2 affinity for PA.

**Table 2:** The predicted differences in binding energies (ΔΔ*G*) of CMG2 variants binding to PA compared to wild-type CMG2.

Mutations of CMG2	ΔΔ*G*
E117Y	−3.61
E117H	−0.55
E117A	−2.66
E117T	−1.76
E117Q	−1.15
E117R	−1.97
E117D	−0.95
Y158M	0.09
Y158W	−0.08
Y158F	−0.05
Y158A	−0.2
Y158G	−0.15
Y158K	0.01
Y158Q	−0.07
Y158N	−0.59

Note: The change in energy is measured in kcal/mol and applies to the change of a single binding interface bound to a PA molecule.

There are no fully automated protein design algorithms that offer a 100% reliable solution to the design process [26]. The user should carefully examine the solutions that are provided by the protein design program.

After studying the calculated binding energy values, a visual inspection of the CMG2-PA model was performed, and the CMG2 variants that were predicted to increase the affinity of CMG2 for PA were selected.

**Figure 1:**
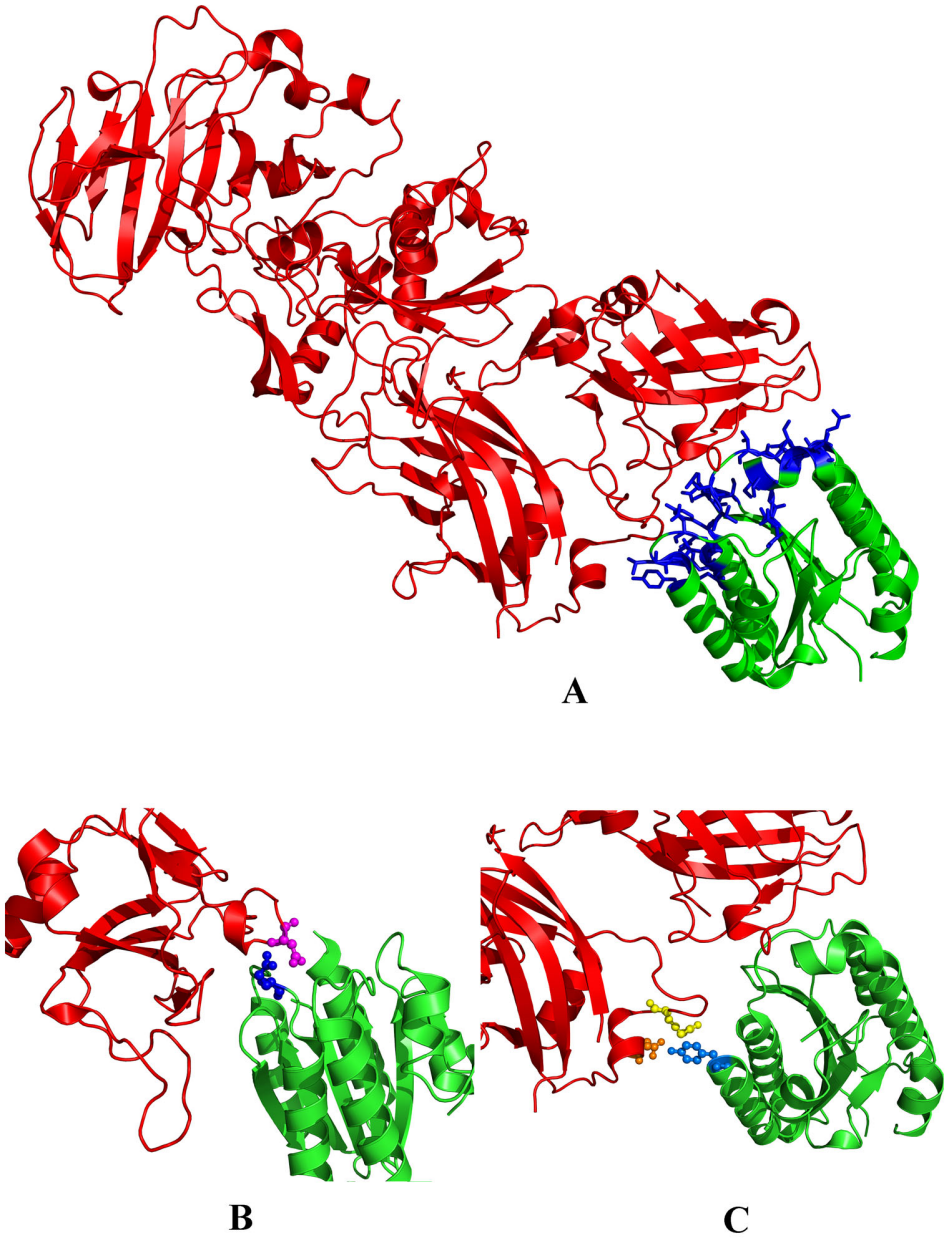
Structure of the CMG2-PA complex. (A) The crystal structure of the CMG2-PA complex (PDB ID: 1T6B). PA is shown in red, CMG2 is shown in green, and the interface residues on CMG2 are shown in blue. (B) Close views of the interactions between PA D683 and CMG2 E117. The two residues are shown as sticks and balls. PA D683 is depicted in yellow, and CMG2 E117 is depicted in blue. (C) Close views of the interactions between PA 344R and CMG2 Y158. The two residues are shown as sticks and balls. PA R344 is depicted in magenta, and CMG2 Y158 is depicted in blue.

From the CMG2-PA complex model, we found that there were some unfavorable interactions between CMG2 and PA. One of these unfavorable interactions was caused by Glu117 of CMG2; Glu117 is located on the area of contact where CMG2 binds to PA and was opposite to the Asp683 residue of PA (Fig. 1b). When the two molecules are in close proximity, a repulsive force may form between these two negatively charged amino acids, which would be unfavorable for CMG2-PA interactions. The substitution of Glu117 with neutral polar or positively charged residues would decrease the repulsive force between the two sites. Because an increase in size of the amino acid would cause steric hindrance between the substituted residue with surrounding ones, in order to avoid causing unexpected steric hindrance, E117 of CMG2 should be mutated to an amino acid that has a similar side chain size to Glu. As an uncharged polar amino acid, Gln differs in charge property, but has similar size relative to Glu. The replacement of Glu117 in CMG2 with Gln may reduce the repulsive interaction with the negatively charged D683 of PA without causing a steric clash. We expected this replacement may improve the binding affinity between the two molecules.

Another unfavorable interaction was caused by Tyr158 of CMG2 (Fig. 1c). Tyr158 is a hydrophobic residue and is located on the interface between CMG2 and PA. It is in close proximity to a hydrophilic region on PA that is comprised of several polar amino acid residues, including Arg344 and Glu348 (Fig. 1c). A substitution of Tyr158 with a polar residue may improve the interaction between CMG2 and PA.

After visual inspection, we modeled the structures for the mutants (E117Q and Y158Q) CMG2 with PA using the build mutant’s protocol of DS 2.5.

**Figure 2:**
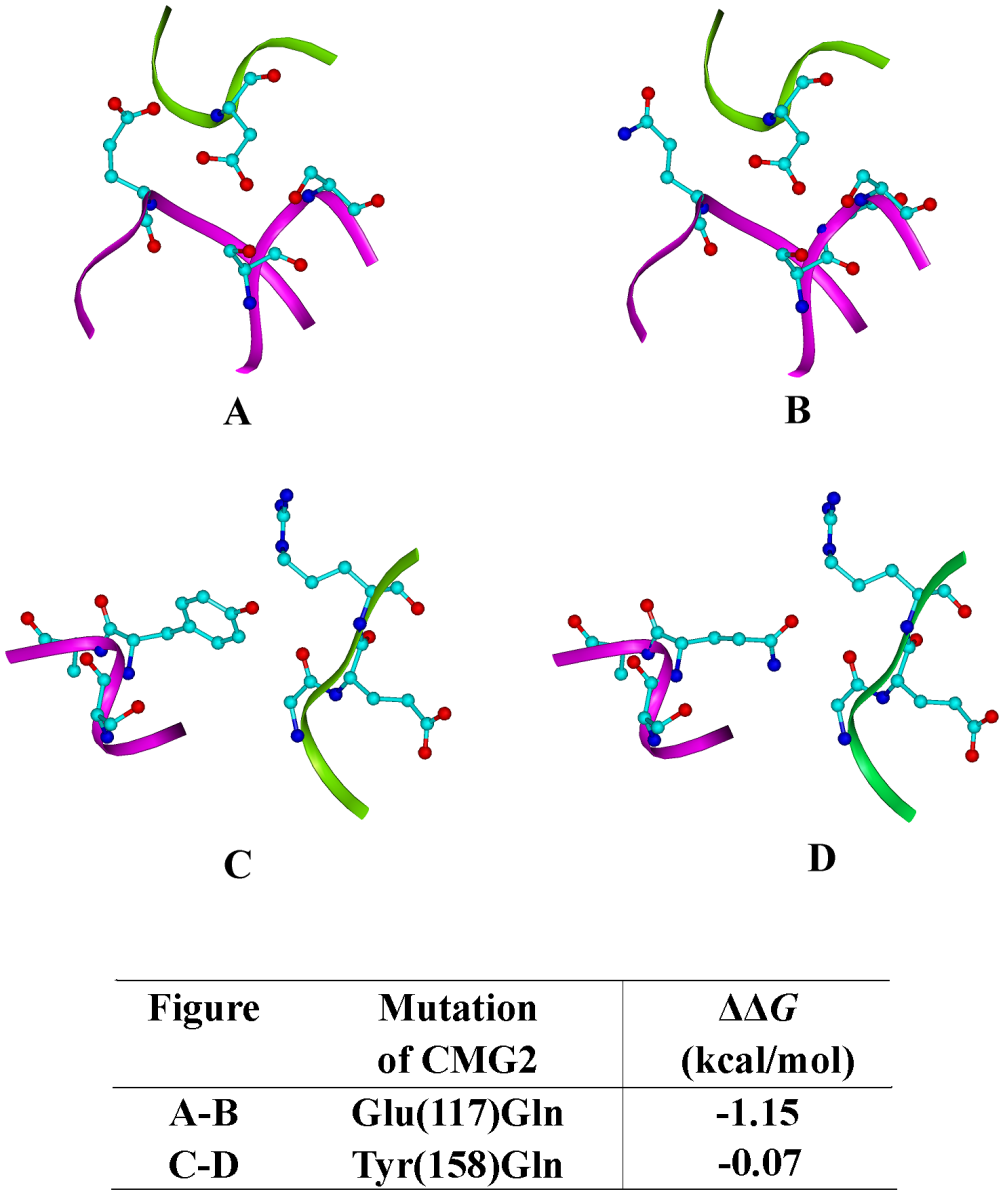
Predicted structures for designed mutations; green ribbon, PA backbone; magenta ribbon, CMG2 backbone. Carbon atom, cyan; Nitrogen atom, blue; Oxygen atom, red. Mutation of CMG2 117 Glu to Gln is shown in (A) and (B), residues 117, 52, 54 and PA 683 are highlighted as sticks and balls. (C) and (D) indicate CMG2 158Tyr to Gln, position 158 of CMG2 and residues 342–344 of PA are highlighted as sticks and balls. Binding energies are given beneath.

In the predicted CMG2(E117Q)-PA complex model, the repulsive electrostatic interaction between Asp683 of PA and position 117 of CMG2 did disappear (Figure 2). Together with diminishing the repulsive interaction, E117Q mutant broke the hydrogen bonding formed between the E117 of CMG2 and the D683 of PA, but the overall effect of the charge inversion at this position gave a positive contribution to the binding between CMG2 and PA. Binding free energy (ΔΔG binding) calculations indicated that this mutation produced a lower binding free energy relative to WT (Figure 2). Overall, substitution in position 117 of CMG2 increases the binding affinity between PA and CMG2.

In the predicted CMG2(Y158Q)-PA complex model, mutation at position 158 of CMG2 make this site more compatible with its environment (Figure 2).

### Protein purification and identification

**Figure 3:**
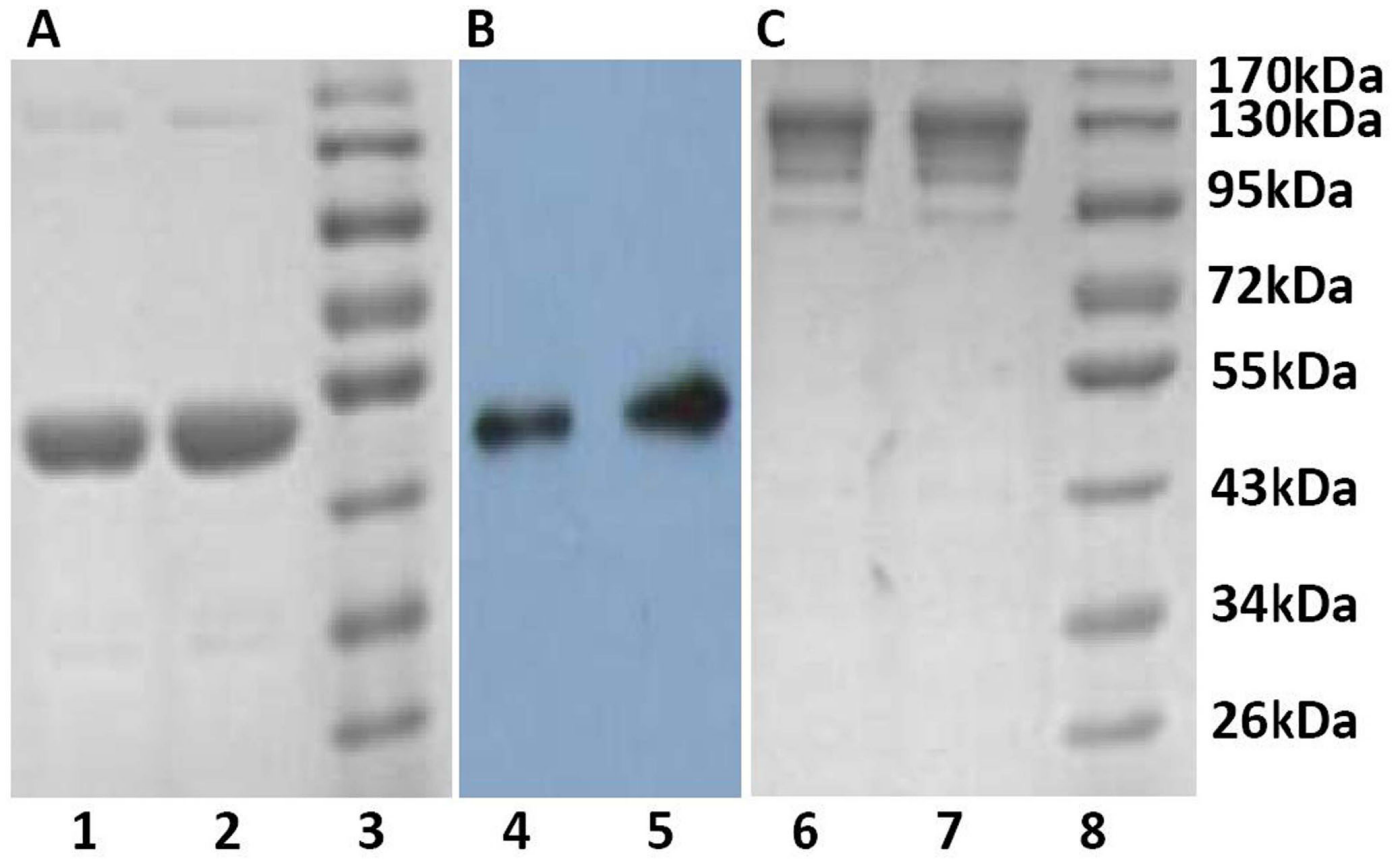
The purified CMG2-Fc mutants were analyzed by 10% SDS-PAGE under reducing conditions (A, B) or non-reducing conditions (C). Gels were visualized by Coomassie blue staining (A, C) or subjected to western blotting with a mouse anti-CMG2 antibody (B). Lanes 1, 4 and 6: CMG2-Fc (Y158Q); lanes 2, 5 and 7: CMG2-Fc (E117Q); lanes 3 and 8: protein marker.

CMG2-Fc is a glycoprotein and must be expressed in eukaryotic cells, such as the CHO cell line. CHO cells are the most dependable eukaryotic host cells for the industrial production of therapeutic proteins due to their appropriate product secretion post-translational processing, a reasonable safety profile and the approval of regulatory authorities [28]. The CHO-S cell line is a stable cell line sub-cloned from the common CHO cells. The most pertinent advantage of this cell line is that it can be adapted to suspension culture and therefore reduce costs. A recombinant CHO-S cell line producing CMG2-Fc mutant was selected for culture in a 500 ml shake flask (Corning) with serum-free DMEM/F12 medium. The proteins were purified by protein A and identified by SDS-PAGE and western blotting (Figure. 3). The CMG2-Fc mutant is comprised of amino acids 1–225 of human CMG2, followed by the hinge and Fc regions of human IgG1 (the C-terminal 232 amino acids of IgG1). The molecular weight of this fusion was approximately 47 kDa under reducing conditions (consistent with CMG2-Fc) as determined by mass spectroscopy (data not shown). The expression of protein was identified by Western blot analysis using mouse anti-CMG2 polyclonal antibody (Anti-ANTXR2 antibody ab70499, Abcam, UK).

### Affinity assay

The affinities of the interactions between the CMG2-Fc mutants and PA were analyzed using the ForteBio QKe and anti-human IgG Fc capture biosensors (AHC sensor). The results (Table 3) showed that the E117Q substitution could improve the binding between CMG2-Fc and PA; the K_D_ of CMG2-Fc (E117Q) for PA is 0.2 nM, which is lower than that of CMG2-Fc (K_D_ = 0.84 nM; p<0.05). The Y158Q substitution also slightly strengthened the binding between CMG2-Fc and PA.

**Table 3:** Kinetic data for the binding of PA with CMG2-Fc mutants.

	K_D_ (nM)	k_on_	k_dis_	*P^a^*
CMG2-Fc(E117Q)	0.20±0.02	19.41±0.70	3.93±0.53	0.000
CMG2-Fc(Y158Q)	0.76±0.05	11.10±0.68	8.47±1.08	0.091
CMG2-Fc	0.84±0.03	11.50±1.46	9.70±1.15	

NOTE: The data represent the mean±SD values of triplicate samples. K_D_, the equilibrium dissociation constant; k_on_ (1/Ms×10^3^), the associated rate constant; k_dis_ (1/s×10^−6^), the dissociation rate constant; nM, nmol/L; s, second.

*P^a^*, for comparisons to the CMG2-Fc group using a Student’s unpaired *t*-test.

### TNA *in vitro*

**Figure 4:**
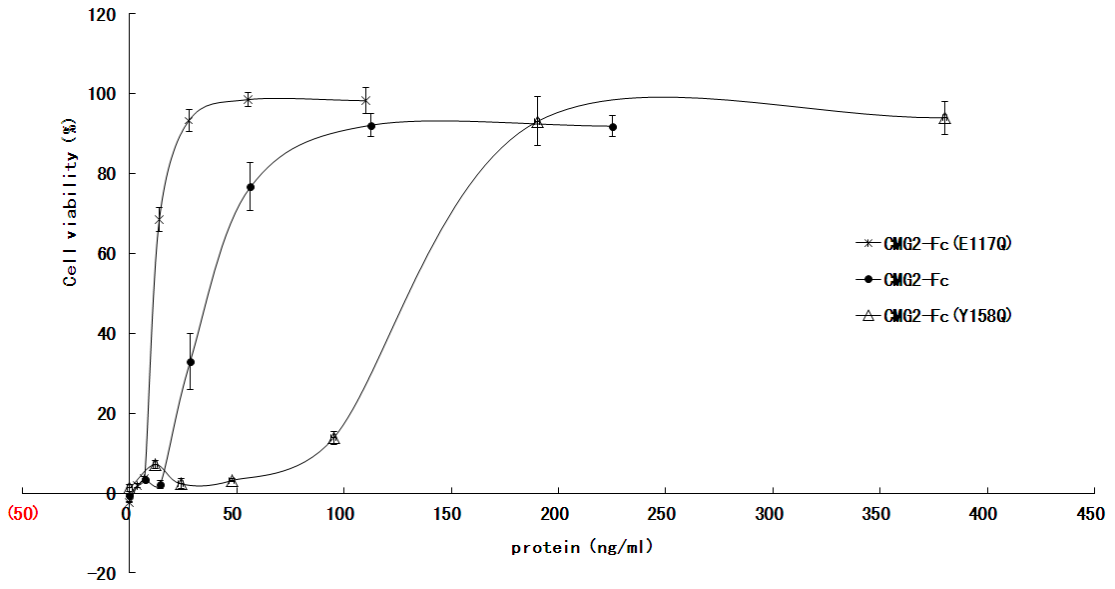
Toxin neutralization assay analyzing the CMG2-Fc mutants. The experiments were performed at different concentrations and dilutions due to the different abilities of the different mutants to neutralize LeTx. The data points represent the mean ± SD values of triplicate samples.

The ability of the CMG2-Fc mutants to neutralize LeTx toxicity in J774A.1 mouse macrophage cells was analyzed over a range of concentrations. The lethal doses in J774A.1 cells were 200 ng of LeTx. For the toxin neutralization assay, we designed different initial dilutions based on the respective EC_50_ calculated by the preliminary assay. Figure 4 shows a typical dose-response curve, from which an EC_50_ can be calculated. The average EC_50_ for CMG2-Fc is 49.2 ng/ml, whereas CMG2-Fc (E117Q) had an EC_50_ of 14.2 ng/ml. The ability of CMG2-Fc (E117Q) to protect J774A.1 cells against LeTx challenge is superior to CMG2-Fc (p<0.05). The neutralization ability of CMG2-Fc (Y158Q) is weaker than that of CMG2-Fc; the EC_50_ of CMG2-Fc is 131 ng/ml.

### LeTx challenge in F344 rats

**Figure 5:**
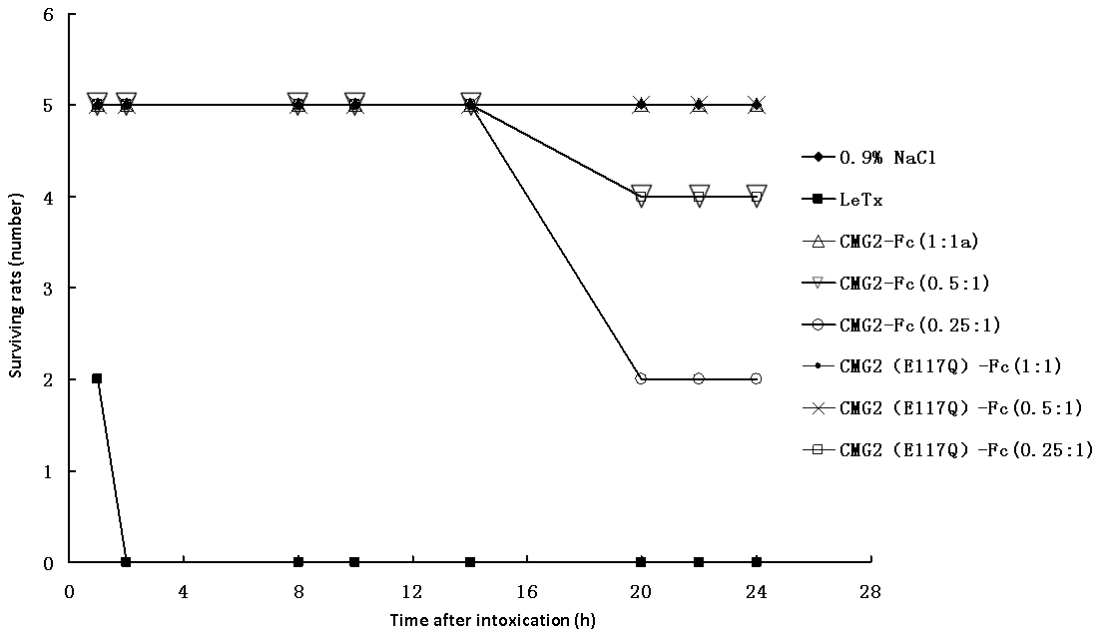
The effect of CMG2-Fc (E117Q) on the survival of F344 rats challenged with lethal toxin (LeTx). The weight ratio of CMG2-Fc to lethal factor was evaluated at 1∶1. The rest may be deduced by analogy.

The ability of CMG2-Fc (E117Q) and CMG2-Fc to neutralize LeTx was compared *in vivo*. Male Fisher 344 rats were injected via the tail vein with LeTx (100 µg PA and 100 µg LF), either alone or in combination with various doses CMG2-Fc (E117Q) (100 µg, 50 µg or 25 µg). The results (Figure 5) show that all of the rats that received only LeTx died within 2 hours; however, for the CMG2-Fc (E117Q) groups, all of the rats receiving LeTx and CMG2-Fc (E117Q) at the mass ratio of 0.5∶1 survived, and only one rat died at the lower ratio of CMG2-Fc(E117Q) to LeTx (0.25∶1). In contrast, three of the rats receiving LeTx and CMG2-Fc at a ratio of 0.5∶1 died, and three of the rats receiving the lower ratio of CMG2-Fc:LeTx (0.25∶1) also died (Figure 5).

## Discussion

Anthrax toxins, the major virulence factors of anthrax, are associated with all stages of the disease; they inhibit the innate immune system, disrupt the adaptive immune system, increase vascular permeability, and lead to septic shock and death. However, anthrax toxins must first be transported into the cytoplasm by toxin receptors (CMG2 or TEM8) before they can cause these deleterious effects.

The CMG2-Fc is a fusion protein consisting of the vWA domain of CMG2 and the Fc region of IgG1. This fusion may extend the biological half-life of CMG2 [14]. CMG2-Fc has high *in vitro* toxin-neutralizing potency and can protect rabbits against lethal pulmonary infection [14]. As a toxin-neutralizing agent, its therapeutic ability can be improved by enhancing the binding affinity of the CMG2-PA interaction.

Computational protein design methods have been demonstrated by others to represent a valuable tool for the improvement and modification of protein-protein interactions [29]–[33]. From a practical point of view, computational design algorithms enable the modification of several key properties of proteins in a much shorter time frame than any other protein engineering methodologies, such as directed evolution methods [24].

In this study, we calculated the binding energies using the FoldX algorithm to determine the residues that may be important for the binding between CMG2 and PA. We used the calculated binding energies to determine the increased or decreased affinities of CMG2 variants for PA, which were verified by our experimental results.

Based on the crystal structure of the CMG2-PA complex, it can be observed that the mutation of residue E117, which is located in the loop α2-α3 of CMG2 and participates in the formation of a hydrophobic ridge structure, strengthens the interaction between CMG2 and PA. However, the FoldX analysis results also indicated that E117 has a second effect on the CMG2-PA interaction: the substitution of E117Q could decrease the repulsive force between the two sites. Subsequent assays confirmed this hypothesis. The affinity results showed that the E117Q mutation obviously strengthened the binding between CMG2 and PA, and the neutralization assays *in vitro* and *in vivo* confirmed that CMG2-Fc (E117Q) could neutralize anthrax toxin and protect F344 rats against lethal toxin challenge; these assays also indicated that CMG2-Fc (E117Q) is preferable to CMG2-Fc and can be used to treat anthrax infection together with other anti-anthrax molecules.

Y158 is the other unfavorable residue for the CMG2-PA interaction, and the affinity assay indicated that the Y158Q mutation slightly strengthened the binding relative to CMG2-Fc; however, the cell protection assay *in vitro* showed that this mutant did not enhance the ability of CMG2-Fc to neutralize anthrax toxin. This may be because one or more molecules secreted by the cells attenuated the interaction between CMG2 (Y158Q) and PA.

In summary, we conducted computational protein design to direct the generation of CMG2-Fc variants with high affinity to PA. The computational method combines automated protein design algorithms with visual inspection, which is demonstrated to be an effective strategy for improving binding affinities. The design predictions for high PA-binding activity variants resulted in variant that shows higher PA-binding ability and toxin-neutralizing potency *in vitro* and *in vivo* than CMG2-Fc. To our knowledge, this work is the first report of the use of a CMG2 mutant as a treatment against anthrax toxin challenge. Additional studies, including the challenge of rabbits with *B. anthracis* spores and pharmacokinetic assays, are ongoing. We explored the possibility of mutating selected amino acids in CMG2 to obtain a CMG2-Fc mutant with higher activity for treatment against anthrax toxin challenge. Our method can be further applied to design other CMG2-Fc mutants with improved therapeutic capacity.
